# Prognostic Impact of MAFLD Following Surgical Resection of Hepatitis B Virus-Related Hepatocellular Carcinoma: A Nationwide Cohort Study

**DOI:** 10.3390/cancers14205002

**Published:** 2022-10-13

**Authors:** Byungyoon Yun, Sang Hoon Ahn, Juyeon Oh, Jin-Ha Yoon, Beom Kyung Kim

**Affiliations:** 1 Department of Preventive Medicine, Yonsei University College of Medicine, Seoul 03722, Korea; 2 Department of Internal Medicine, Yonsei University College of Medicine, Seoul 03722, Korea; 3 Institute of Gastroenterology, Yonsei University College of Medicine, Seoul 03722, Korea; 4 Yonsei Liver Centre, Severance Hospital, Yonsei University Health System, Seoul 03722, Korea; 5 Department of Public Health, Yonsei University Graduate School, Seoul 03722, Korea; 6 The Institute for Occupational Health, Yonsei University College of Medicine, Seoul 03722, Korea

**Keywords:** hepatitis B virus, hepatocellular carcinoma, MAFLD, surgery, prognosis

## Abstract

**Simple Summary:**

We examined the relationship between metabolic dysfunction-associated fatty liver disease (MAFLD) and the recurrence of hepatocellular carcinoma in patients treated by surgical resection of a primary hepatitis B virus-related HCC. MAFLD was significantly associated with 22% and 44% increased risk of HCC recurrence and all-cause mortality, respectively. This study was undertaken to underline the need for developing effective preventive strategies through the management of metabolic health to reduce the incidence of MAFLD in the population.

**Abstract:**

The association between the metabolic effects of hepatic steatosis as a part of postoperative outcomes of hepatitis B virus (HBV)-related hepatocellular carcinoma (HCC) has rarely been studied. This study aimed to assess the relationship between metabolic dysfunction-associated fatty liver disease (MAFLD) and patients’ prognoses following curative resection of HBV-related HCC. Patients who underwent surgical resection for HBV-related HCC between 2009 and 2015 were recruited. The study endpoints were postoperative hepatocellular carcinoma (HCC) recurrence and all-cause mortality. Adjusted hazard ratios (aHRs) for the outcomes were estimated using multivariate Cox regression models. The mean age of the 2032 enrolled patients was 55.0 years, and 77.9% were men. During follow-up (median 5.3 years), HCC recurrence and all-cause mortality occurred in 954 (47.0%) and 422 (20.8%) patients, respectively. HCC recurrence and all-cause mortality were significantly associated with MAFLD, with aHRs of 1.22 (*p* = 0.003) and 1.44 (*p* < 0.001), respectively. Propensity score matching and inverse probability treatment weighting analyses confirmed similar results (*p* < 0.05). MAFLD was associated with significantly poor prognoses in terms of HCC recurrence and all-cause mortality following surgical resection of HBV-related HCC. Further studies are needed to develop an effective preventive strategy through the management of metabolic health.

## 1. Introduction

Hepatitis B virus (HBV) infection affects approximately 300 million people worldwide and is a significant public health concern; the disease burden is particularly significant in HBV-endemic regions, including East Asian countries [1,2,3,4]. Hepatocellular carcinoma (HCC) is commonly caused by HBV and is the second leading cause of global cancer-related mortality [5]; therefore, a comprehensive prevention and treatment strategy for HCC is greatly needed. Long-term antiviral therapy (AVT), with potent oral nucleos(t)ide analogs (NUCs), significantly decreases the risk of HCC and/or liver cirrhosis (LC), effectively controlling the high viral load, the most important risk factor for HCC [6,7,8]. Interestingly, several studies note that metabolic diseases, including diabetes, obesity, and non-alcoholic fatty liver disease (NAFLD), are significant risk factors for HCC [9,10,11].

Although surgical resection is considered a curative treatment for HCC, its recurrence is a significant challenge, with a 5-year postoperative recurrence rate >50% [12,13,14]. Prophylactic use of oral NUCs, may reduce the risk of HBV-related HCC recurrence and mortality [15,16]. As a high HBV-DNA level is related to a higher rate of postoperative HCC recurrence [17], patient metabolic parameters should be examined as risk factors for postoperative HCC recurrence and mortality.

The prevalence of poor metabolic health has increased worldwide [18], even among normal-weight populations [4,19,20,21]. Metabolic dysfunction-associated fatty liver disease (MAFLD) was recently defined as a more accurate description of hepatic disorders with dynamic interactions among environmental and genetic factors and metabolic syndrome components [22,23,24]. Numerous studies have assessed the relationship between MAFLD and mortality or the risk of cardiovascular disease [25,26]. However, the relationship between HCC incidence and postoperative HCC recurrence has not been clinically validated.

This study aimed to assess the association of postoperative HCC recurrence and all-cause mortality with MAFLD in patients with HBV-related HCC, independent of other demographic, virological, and environmental factors, using a nationwide health insurance system (NHIS) database.

## 2. Materials and Methods

### 2.1. Data Source

This nationwide retrospective cohort study used data for enrolled participants gathered from the National Health Insurance Service in the Republic of Korea. Data on baseline characteristics, diagnostic codes, prescription history, procedural codes, and health examination results of patients with HCC were extracted from the entire database. Data of patients (aged ≥20 years) with chronic HBV infection who underwent curative surgical resection for HCC between January 2009 and December 2015 were collected. The index date was the exact date of the operation. Individuals who participated in health examination within ±2 years from the index date were initially recruited. The health examination data closest to the index date was used. Health examination data includes a standardized questionnaire regarding lifestyle factors (smoking, alcohol consumption, and physical activity), body measurements (body mass index (BMI), waist circumference (WC), and blood pressure), and laboratory data (fasting glucose, liver enzymes, and cholesterol profiles).

Patients were excluded according to the following criteria: (1) missing values in health examination records, including components to calculate the fatty liver index (FLI); (2) history of human immunodeficiency virus or hepatitis C virus infection before the index date; (3) history of extrahepatic malignancy before the index date; (4) concurrent procedures (radiation therapy, local ablation, chemoembolization, and systemic chemotherapy) against HCC at the same index date; and (5) orthotopic liver transplantation (OLT), radiation therapy, systemic chemotherapy, or death within 3 months after enrolment. All diseases were defined according to the International Classification of disease, tenth edition codes (ICD-10), with the criteria of three or more outpatient visits or one or more hospitalizations (Supplementary Table S1). According to the practice guideline in South Korea, participants were followed-up for post-resection HCC recurrence at 3–6 months interval based upon laboratory tests, including tumor markers and computed tomography and/or magnetic resonance imaging [13,27].

This study was approved by our institutional review board and has been conducted in accordance with the ethical requirements of the 1975 Declaration of Helsinki (IRB 4-2020-1213). The need for informed consent was waived owing to the retrospective nature of the study.

### 2.2. Outcomes of the Study

The primary outcome of this study was the postoperative HCC recurrence. All-cause mortality was considered a secondary outcome. The HCC recurrence date was defined as the first date of any procedure or drug use relevant to curative or palliative treatment after surgical resection, including cryotherapy, local ablation, chemoembolization, radiotherapy, systemic chemotherapy, OLT, and surgical resection [14,28,29]. Patients were followed up until HCC recurrence, death, or December 2019, whichever occurred first.

### 2.3. Definition of Variables

MAFLD was defined as the presence of hepatic steatosis, with a FLI ≥ 30 and one or more of the following criteria: (1) body mass index (BMI) ≥ 23 kg/m^2^ (overweight or obese); (2) diabetes mellitus (Type 2); and (3) presence of ≥2 metabolic abnormalities (Supplementary Table S2) [22].

FLI was calculated through a complex formula of four variables: BMI, waist circumference, triglyceride, and gamma-glutamyl transpeptidase [30]. Hypertension, diabetes mellitus, dyslipidemia, LC, and other diseases used for exclusion were defined based on ICD-10 codes. Medication history, including AVT against chronic HBV infection, metformin, and statins, was defined based on the criterion of the total sum of prescription ≥90 days.

Individuals were classified as non-smokers, former smokers, or current smokers using data based on smoking history and obtained from examination of health records. With respect to alcohol use history, heavy drinkers were defined as male and female participants who consumed 20 g and 10 g of alcohol per day, respectively; moderate drinkers were defined as male and female participants who consumed 1–19 g and 1–9 g of alcohol per day, respectively; and non-drinkers were defined as those who consumed <1 g of alcohol per day [31]. Each participant’s metabolic equivalent of tasks (METs)-hour/week was estimated by adding their history of strenuous (7 METs) and moderate (4 METs) physical activity, as well as walking (2.9 METs) [32]. Participants’ physical activity was classified into four groups based on cut-off values of 3, 9, and 18 METs-hour/week [33]. Obesity was classified into four categories based on Asian BMI guidelines: underweight (18.5 kg/m^2^), normal weight (18.5–22.9 kg/m^2^), overweight (23–24.9 kg/m^2^), and obese (25 kg/m^2^) [34].

### 2.4. Statistical Analysis

For categorical and continuous variables, the chi-square test and independent *t*-test were employed to assess differences between the MAFLD and non-MAFLD groups. The cumulative incidences of HCC recurrence and all-cause mortality between the two groups were examined using the Kaplan–Meier method and compared using the log-rank test. Multivariate Cox proportional hazard models were used to estimate adjusted hazard ratios (HRs) and 95% confidence intervals (CIs) for outcomes. We also computed sub-distribution HRs and 95% CIs using Fine and Gray regression (FGR), considering all-cause mortality as a competing risk event.

Additionally, we used propensity score (PS) matching with a ratio of 1:1 and a caliper width of 0.1 for the nearest neighbor method to avoid confounding bias. The absolute standardized mean difference was determined to determine the covariate balance. Similarly, inverse probability treatment weighting (IPTW) was used to account for confounding bias between the two groups.

All statistical tests were two-sided, and a *p*-value < 0.05 was considered significant. SAS Enterprise version 7.1 (SAS Institute, Cary, NC, USA) and R software version 4.0.3 (R Foundation for Statistical Computing, Vienna, Austria) were used for all the statistical analyses.

## 3. Results

### 3.1. Baseline Characteristics of Patients

After exclusion, 2032 patients were included in the study (Supplementary Figure S1). The mean (standard deviation [SD]) age was 55.0 (8.6) years, and 77.9% were men. The baseline characteristics of the patients, stratified by MAFLD, are summarized in Table 1. Of 2032 patients, 1576 patients received health examinations before surgical resection, while 456 patients received health examination after surgical resection. The MAFLD group (n = 888) had a significantly higher prevalence of male sex, hypertension, diabetes, dyslipidemia, metformin and statin use, current smoking, alcohol consumption, and physical inactivity than the non-MAFLD group (n = 1144; all *p* < 0.05).

Furthermore, 26 and 28 patients in the non-MAFLD and MAFLD groups were treated with additional TACE within 3 months after surgical resection (*p* = 0.278), respectively and four and two patients in the non-MAFLD and MAFLD groups were treated with additional RFA within 3 months after surgical resection (*p* = 0.703), respectively.

### 3.2. Clinical Outcomes of HCC Recurrence and All-Cause Mortality between the MAFLD and Non-MAFLD Groups in the Entire Cohort

The median follow-up duration was 5.3 years (IQR 1.7–8.5). During follow-up, HCC recurrence occurred in 954 (47.0%), 447 (50.3%), and 507 (44.3%) patients in the MAFLD and non-MAFLD groups, respectively. All-cause mortality occurred in 422 (20.8%) patients: 218 (24.6%) in the MAFLD group and 204 (17.8%) in the non-MAFLD group.

**Figure 1:**
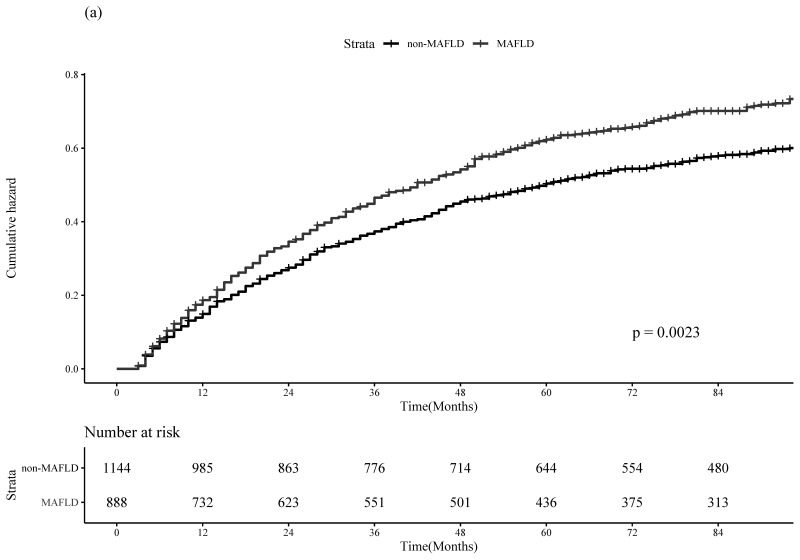

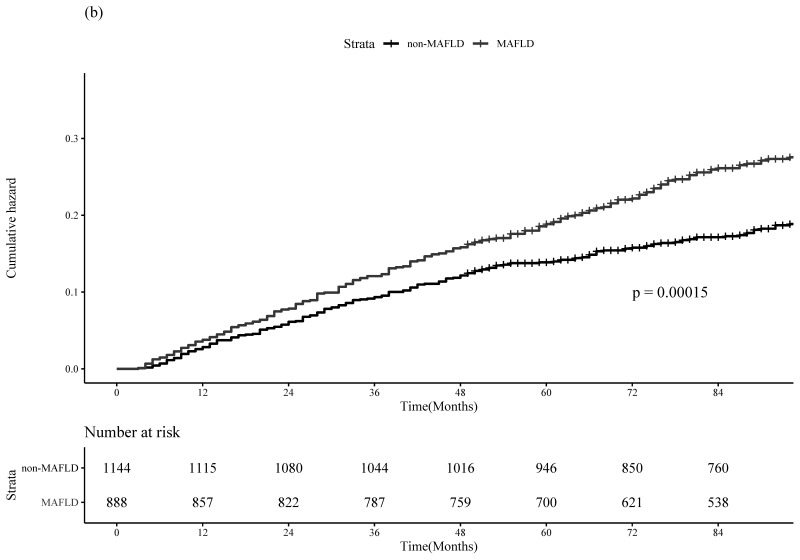
Cumulative incidence plots of postoperative HCC recurrence (**a**) and all-cause mortality (**b**) stratified by MAFLD among the entire cohort.

The MAFLD group had significantly higher incidence of 1-, 3-, and 5-year HCC recurrence than the non-MAFLD group [17.0%, 37.2%, and 46.4% in the MAFLD group vs. 13.9%, 31.2%, and 39.6% in the non-MAFLD group, respectively; *p* = 0.002)] (Figure 1a). The MAFLD group also had significantly higher incidence of 1-, 3-, and 5-year all-cause mortality than the non-MAFLD group [3.7%, 11.4%, and 17.2% in the MAFLD group vs. 2.8%, 8.9%, and 13.0% in the non-MAFLD group, respectively; *p* < 0.001] (Figure 1b). The HRs (95% CIs) of HCC recurrence and all-cause mortality by MAFLD were 1.22 (1.07–1.38; *p* = 0.003) and 1.44 (1.19–1.75; *p* < 0.001), respectively.

According to multivariable Cox regression analysis adjusted for age, sex, LC, metformin and statin use, AVT, smoking history, alcohol consumption, and physical activity, the MAFLD group was more likely to have a higher risk of HCC recurrence with an adjusted HR of 1.20 (95% CI 1.04–1.37; *p* = 0.01) and all-cause mortality with an adjusted HR of 1.44 (95% CI, 1.17–1.76; *p* = 0.001) (Table 2). The full version of Cox regression model is summarized in Supplementary Table S3.

Moreover, considering all-cause mortality as a competing risk in the FGR model, HCC recurrence was significantly associated with MAFLD with a sub-distribution HR of 1.18 (95% CI, 1.03–1.35; *p* = 0.015).

### 3.3. PS-Matching and IPTW Analyses

**Figure 2:**
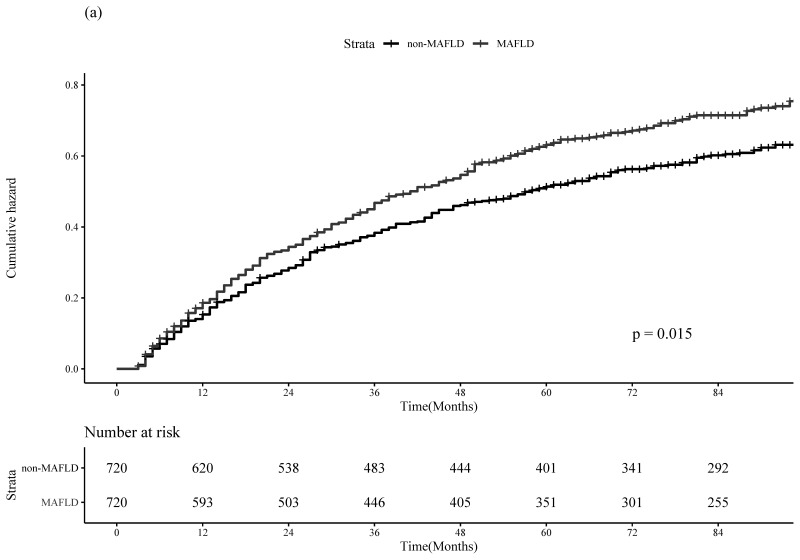

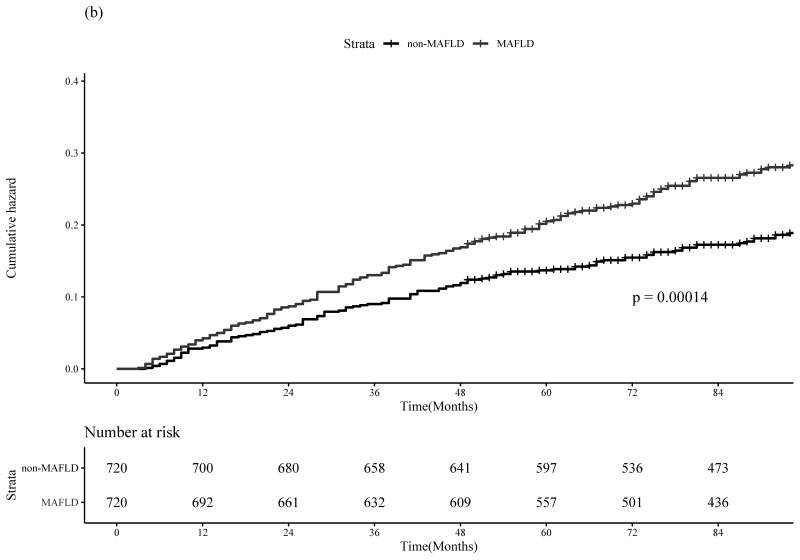
Cumulative incidence plots of postoperative HCC recurrence (**a**) and all-cause mortality (**b**) stratified by MAFLD after PS-matching analysis.

The PS was calculated using a logistic regression model based on age, sex, LC, metformin use, statin use, AVT, smoking history, alcohol consumption, and physical activity. After performing PSM with absolute standardized mean differences below 0.1 for all variables (Supplementary Figure S2), 720 pairs of patients were included in the analysis. According to the baseline characteristics of the MAFLD and non-MAFLD groups in the matched cohort, there was no significant difference between the two groups, except for hypertension and diabetes (all *p* > 0.05; Supplementary Table S4). The MAFLD group had significantly higher 1-, 3-, and 5-year cumulative incidences of HCC recurrence in the matched cohort than the non-MAFLD group [17.0%, 37.4%, and 46.8% in the MAFLD group vs. 14.2%, 31.9%, and 40.2% in the non-MAFLD group; *p* = 0.015)] (Figure 2a). The MAFLD group also had significantly higher 1, 3, and 5-year cumulative incidences of all-cause mortality than the non-MAFLD group [4.2%, 12.2%, and 18.5% in the MAFD group vs. 2.9%, 8.6%, and 12.8% in the non-MAFLD group; *p* < 0.001] (Figure 2b).

Multivariable Cox analysis of PS-matched cohort after adjustment for age, sex, LC, metformin, statin use, antiviral therapy, smoking, alcohol consumption, physical activity also showed significant results with adjusted HRs of 1.22 (95% CI 1.05–1.42; *p* = 0.01) for HCC recurrence and 1.52 (95% CI 1.21–1.91; *p* < 0.001) for all-cause mortality (Table 3). Likewise, when considering all-cause mortality as a competing risk with the FGR model, HCC recurrence was significantly associated with MAFLD (sub-distribution HR 1.20, 95% CI 1.04–1.40; *p* = 0.014).

Furthermore, IPTW analysis showed similar findings. Multivariable Cox analysis also confirmed that the MAFLD group had a significantly higher risk of HCC recurrence with an adjusted HR of 1.26 (95% CI 1.16–1.36; *p* < 0.001) and all-cause mortality with an adjusted HR of 1.44 (95% CI, 1.28–1.61; *p* <0.001) (Table 3).

### 3.4. Sensitivity Analyses

For sensitivity analysis, patients were stratified into three groups with an FLI score cut-off of 30 and 60 (0 to <30; Grade 0 (G0), 30 to <60; Grade 1 (G1), ≥60; Grade 2 (G2)). Patients with FLI G2 were at a higher risk of HCC recurrence with an adjusted HR of 1.37 (95% CI 1.13–1.65; *p* = 0.001) than those with FLI G0 to 1, and patients with G1 to 2 were at a higher risk of all-cause mortality with an adjusted HR of 1.82 (95% CI 1.39–2.39; *p* < 0.001) than patients with FLI G0 (Supplementary Table S5).

According to the proportion of unhealthy lifestyle among individuals receiving health examination before or after surgical resection, “after surgical resection” group presented considerably higher proportion of healthy lifestyle, compared to “before surgical resection” group (Supplementary Table S6). Multivariable Cox regression analysis only for individuals who received health examination before surgical resection also reproduced the result of same direction with an adjusted HR of 1.20 (95% CI 1.02–1.40; *p* = 0.026, Supplementary Table S7).

With different cut-off of FLI with 60, MAFLD was still significantly associated with the increased risk of HCC recurrence. Likewise, FLI cut-off of 31 for men and 18 for women also reproduced the similar result. Furthermore, without adjustment for metformin and statin, the result presented the significant relationship between MAFLD and increased risk of HCC recurrence. Finally, after excluding patients receiving additional TACE within 3 months after surgical resection, the association between the increased risk of HCC recurrence and MAFLD was significant. Likewise, after excluding patients receiving RFA within 3 months after surgical resection, the same result was reproduced (all *p* < 0.05; all Supplementary Table S7).

## 4. Discussion

The study results indicate that MAFLD is significantly associated with HCC recurrence and all-cause mortality. Multivariate Cox analysis adjusted for age, sex, LC, metformin and statins use, AVT, smoking, alcohol consumption, and physical activity demonstrated similar findings. Moreover, the association remained significant across various statistical methods, including PS-matching and IPTW.

Several studies examining the association of fatty liver disease and/or metabolic dysfunction with the risk of HCC development among patients with chronic HBV infection have reported inconsistent conclusions; some large retrospective cohort studies elucidated positive correlations [11,35,36], while others found no significant associations [37,38]. However, there is an evident lack of studies on fatty liver and HCC recurrence, especially after surgical resection of HBV-related HCC. A meta-analysis of 9 studies between the NAFLD etiologies and disease-free survival or overall survival after curative resection for HCC showed that NAFLD has paradoxically reverse associations with disease-free survival and overall survival, compared to non-NAFLD with HRs of 0.85 (95% CI 0.74–0.98; *p* = 0.03) and 0.87 (0.81–0.93; *p* < 0.001), respectively [39]. However, our multimodal analysis consistently indicated that MAFLD is significantly associated with increased risk of HCC recurrence and all-cause mortality.

There are several plausible explanations for these results. Lipid accumulation in hepatocytes can cause fibrogenic activation and even hepato-carcinogenesis through c-Jun N-terminal kinase/activator protein-1 activity or the nuclear factor kappa B pathway [9]. Additionally, excess adipose tissue may also activate the Janus kinase pathway, promoting the production of pro-inflammatory and pro-fibrogenic cytokines, resulting in increased leptin and decreased adiponectin [40,41]. Yoshimoto et al. found that a bacterial metabolite (deoxycholic acid)-induced senescence-associated secretory phenotype could mediate hepatic stellate cell activation and lead to hepatocarcinogenesis [42]. In line with these theoretical backgrounds, the severity of hepatic steatosis measured by FLI was also significantly associated with HCC recurrence and all-cause mortality in a dose-dependent manner. Notably, the harmful effect of hepatic steatosis on HCC recurrence became evident only with FLI ≥ 60; its effect on all-cause mortality became evident in the earlier phase of hepatic steatosis when the FLI was ≥30. This indirectly supports the hypothesis that hepatic steatosis affects liver-related outcomes as well as non-liver-related outcomes, such as death from cardiovascular disease.

Our study has several strengths. First, through a nationwide cohort, we enrolled a large sample size (n = 2032) of patients with HBV-related HCC who underwent curative surgical resection. This study also examined the characteristics of the enrolled patients, including demographic factors, past disease history, medication history, and lifestyle factors. Second, various statistical analyses, including FGR, PSM, and IPTW, were performed to minimize unmeasured confounding bias due to the incompleteness of the nationwide data. Third, to the best of our knowledge, this is the first study to examine the association of MAFLD with postoperative HCC recurrence and all-cause mortality in patients with chronic HBV infection. In this study, 43.7% (888 of 2032) of all patients with HBV who underwent HCC resection were affected by MAFLD, which is much higher than the prevalence in the general population (37.3%) in the Republic of Korea [26]. Our findings suggest MAFLD as a risk factor for HCC recurrence and further studies should be conducted to better represent the true population and corroborate these results.

This study had several limitations. First, owing to the inherent limitation of the NHIS database, the exact data concerning tumor size and number, other histological data (e.g., R0 resection, microvascular invasion, tumor grade, etc.), serum HBV-DNA level or other serological markers, and radiological evidence of HCC recurrence were missing [43,44,45,46]. To minimize some of such bias, when we excluded patients receiving additional therapy (i.e., RFA and/or TACE) within 3 months after the surgical resection, the similar results were observed. Furthermore, we can cautiously speculate that at least a limitation regarding serum HBV-DNA level might be partially overcome, considering the stringent reimbursement guideline of oral NUCs by the NHIS (serum HBV-DNA ≥ 20 IU/mL) [8]. Concerning the definition of recurrence with any kind of recurrence treatment, due to lack of hospital-level information, patients not receiving any antitumor therapy due to bad performance state could have been missed based on this definition of recurrence. Nonetheless, given that the proportion of patients treated with only best supportive care for post-operative recurrence was quite low (5~7%) owing to the bad performance status or insufficient hepatic function to tolerate surgical resection [47,48,49], we might carefully speculate that our operational definition could cover the recurrence appropriately via our large sample size cohort. Further prospective cohort studies with detailed information should be conducted to overcome this limitation. Second, owing to a lack of biopsy results, hepatic steatosis was characterized using FLI. However, FLI, as a surrogate biochemical score, has a high diagnostic performance, with an area under the curve value of 0.86 for males and 0.91 for women [30,50]. We also performed sensitivity analysis with a different cut-off of FLI to validate our result. Third, adjusting metformin and statin might result in indication bias, since the majority of users are patients with diabetes or dyslipidemia. However, additional analysis without adjustment for metformin and statin reproduced the similar significant result. Lastly, the majority of patients in the Republic of Korea were infected with HBV genotype C2 via vertical transmission, which was associated with an increased risk of disease progression [51]. Further studies are required to validate this hypothesis.

## 5. Conclusions

In conclusion, MAFLD was significantly associated with an increased risk of HCC recurrence and all-cause mortality after surgical resection of HBV-related HCC. As MAFLD represents a potentially significant risk factor for postoperative prognoses, further studies are needed for a parallel evaluation with other risk factors and develop an effective preventive strategy through the management of patient metabolic health.

## Figures and Tables

**Table 1:** Baseline Characteristics of Chronic HBV participants stratified by MAFLD.

	The Entire Cohort (n = 2032)		
	Non-MAFLD (n = 1144)	MAFLD (n = 888)	*p*-Value
Age			0.355
	55.1 (8.8)	54.8 (8.4)	
Sex			<0.001
Male	788 (68.9%)	795 (89.5%)	
Female	356 (31.1%)	93 (10.5%)	
Hypertension			<0.001
No	768 (67.1%)	448 (50.4%)	
Yes	376 (32.9%)	440 (49.6%)	
Diabetes			<0.001
No	900 (78.7%)	572 (64.4%)	
Yes	244 (21.3%)	316 (35.6%)	
Dyslipidemia			0.007
No	871 (76.1%)	629 (70.8%)	
Yes	273 (23.9%)	259 (29.2%)	
Liver cirrhosis			0.955
No	407 (35.6%)	317 (35.7%)	
Yes	737 (64.4%)	571 (64.3%)	
Metformin use			<0.001
No	1013 (88.6%)	697 (78.4%)	
Yes	131 (11.4%)	191 (21.5%)	
Statin use			<0.001
No	1009 (88.2%)	697 (78.5%)	
Yes	135 (11.8%)	191 (21.5%)	
Antiviral therapy			0.292
No	243 (21.2%)	206 (23.2%)	
Yes	901 (78.8%)	682 (76.8%)	
Smoking history			<0.001
Non-smoker	564 (49.3%)	296 (33.3%)	
Ex-smoker	303 (26.5%)	274 (30.9%)	
Current smoker	277 (24.2%)	318 (35.8%)	
Alcohol drink			<0.001
None	805 (70.4%)	405 (45.6%)	
Moderate	247 (21.6%)	276 (31.1%)	
Heavy	92 (8.0%)	207 (23.3%)	
Physical activity			0.037
0 to <3 METs-hour/week	344 (30.1%)	297 (33.4%)	
3 to <9 METs-hour/week	272 (23.8%)	202 (22.8%)	
9 to <18 METs-hour/week	330 (28.8%)	273 (30.7%)	
≥18 METs-hour/week	198 (17.3%)	116 (13.1%)	

Abbreviation: MAFLD, metabolic dysfunction-associated fatty liver disease; HBV, hepatitis B virus.

**Table 2:** Adjusted HRs and 95% CIs of HCC recurrence and all-cause mortality by MAFLD in chronic HBV infection.

	HCC Recurrence		All-Cause Mortality	
	Adjusted HR (95% CI)	*p*-Value	Adjusted HR (95% CI)	*p*-Value
MAFLD				
No	1.00 (reference)		1.00 (reference)	
Yes	1.20 (1.04–1.37)	0.01	1.44 (1.17–1.76)	0.001

Abbreviation: HR, hazard ratio; CI, confidence interval; HCC, hepatocellular carcinoma; MAFLD, metabolic dysfunction-associated fatty liver disease; HBV, hepatitis B virus.

**Table 3:** Multivariable analyses of HCC recurrence and all-cause mortality according to MAFLD among patients with HBV-related HCC receiving surgical resection after PS-matching or IPTW.

	MAFLD	HCC Recurrence		All-Cause Mortality	
		Adjusted HR (95% CI)	*p*-Value	Adjusted HR (95% CI)	*p*-Value
PS-Matching	No	1.00 (reference)		1.00 (reference)	
	Yes	1.22 (1.05–1.42)	0.01	1.52 (1.21–1.91)	<0.001
IPTW	No	1.00 (reference)		1.00 (reference)	
	Yes	1.26 (1.16–1.36)	<0.001	1.44 (1.28–1.61)	<0.001

Abbreviation: HCC, hepatocellular carcinoma; MAFLD, metabolic dysfunction-associated fatty liver disease; HBV, hepatitis B virus; PS, propensity score; IPTW, inverse probability treatment weighting, HR; hazard ratio; CI, confidence interval.

## Data Availability

The data in this study were gathered from National Health Insurance Service in the Republic of Korea. Thus, the data cannot be shared publicly without permission.

## Supplementary Materials

The following supporting information can be downloaded at: https://www.mdpi.com/article/10.3390/cancers14205002/s1, Table S1: ICD-10 code used for defining disease or procedure in the study; Table S2: Definition of Metabolic Dysfunction-Associated Fatty Liver Disease; Table S1: title; Table S3: Multivariable Cox regression model of HCC recurrence and all-cause mortality in chronic HBV infection; Table S4: Comparison of baseline characteristics according to MAFLD among patients with HBV-related HCC receiving surgical resection after PS-matching; Table S5: Adjusted HRs and 95% CIs of HCC recurrence by FLI grade; Table S6: The proportion of unhealthy lifestyle among individuals receiving health examination before or after the resection; Table S7: The result of sensitivity analysis on the risk of HCC recurrence by MAFLD; Figure S1: Detailed schematic flow of patient selection; Figure S2: Absolute standardized mean difference of the variables before and after matching.
