# Supplementary material for: Prognostic Impact of MAFLD Following Surgical Resection of Hepatitis B Virus-Related Hepatocellular Carcinoma: A Nationwide Cohort Study
Source: Cancers (Basel). 2022 Oct 13;14(20):5002. doi: 10.3390/cancers14205002 (PMC9599346; doi:10.3390/cancers14205002)
Supplement: Supplementary file 1 [file cancers-14-05002-s001.zip › Suppl Tables S1-S7.pdf]

**Supplementary Table S1. ICD-10 code used for defining disease or procedure in the study**

| **Diagnosis** | **ICD10 code** |
|---|---|
| Hypertension | I10-15 |
| Diabetes | E10-14 |
| Dyslipidemia | E78 |
| Liver cirrhosis | K741,K742,K744,K745,K746 |
| Chronic hepatitis B virus | B180, B181 |
| Chronic hepatitis C virus | B182 |
| Hepatocellular carcinoma | C220 |
| Human immunodeficiency virus | B20-B24 |

**Supplementary Table S2. Definition of Metabolic Dysfunction-Associated Fatty Liver Disease**

**- Existence of hepatic steatosis * with one or more of the following criteria**

(1) Overweight or obesity (Body Mass Index 23 kg/m$^2$ or more)

(2) Type 2 Diabetes

(3) Presence of at least two metabolic abnormalities

Waist circumference 90cm or more for men and 80cm or more for women

Blood pressure >= 130/85mmHg or specific drug treatment

Plasma triglyceride>=150mg/dL or specific drug treatment

Plasma HDL-cholesterol <40mg/dL for men and <50mg/dL for women or specific drug treatment

Prediabetes (fasting glucose levels 100-125mg/dL or 2hour post-prandial glucose levels 140-199mg/dL or HbA1c 5.7%-6.4%)

Homeostatis model assessment of insulin resistance score >=2.5

Plasma high-sensitivity C-reactive protein level > 2mg/L

* hepatic steatosis was defined as fatty liver index (FLI) >=30

* FLI = 1/(1+e-x) * 100; x= 0.593 * loge(triglyceride) + 0.139 * body mass index + 0.718 * loge(gamma-glutamyl-transferase)+0.053*waist circumference -15.745

¶ 2 hour post-prandial glucose levels, HbA1c, homeostasis model assessment of insulin resistance score, and plasma high-sensitivity C-reactive protein level were not eligible due to lack of information

**Supplementary Table S3. Multivariable Cox regression model of HCC recurrence and all-cause mortality in chronic HBV infection**

| | HCC recurrence | | All-Cause Mortality | |
|---|---|---|---|---|
| **Variable** | **Adjusted HR (95% CI)** | **p-value** | **Adjusted HR (95% CI)** | **p-value** |
| **MAFLD** | | | | |
| No | 1.00 (reference) | | 1.00 (reference) | |
| Yes | 1.20 (1.04-1.37) | 0.01 | 1.44 (1.17-1.76) | 0.001 |
| **Age** | 1.01 (1.00-1.02) | 0.024 | 1.02 (1.01-1.03) | 0.006 |
| **Sex** | | | | |
| Women | 1.00 (reference) | | 1.00 (reference) | |
| Men | 1.15 (0.95-1.40) | 0.156 | 1.07 (0.80-1.43) | 0.669 |
| **LC** | | | | |
| No | 1.00 (reference) | | 1.00 (reference) | |
| Yes | 1.51 (1.31-1.75) | <0.001 | 2.03 (1.61-2.59) | <0.001 |
| **Metformin use** | | | | |
| No | 1.00 (reference) | | 1.00 (reference) | |
| Yes | 1.10 (0.92-1.32) | 0.299 | 1.22 (0.94-1.57) | 0.133 |
| **Statin use** | | | | |
| No | 1.00 (reference) | | 1.00 (reference) | |
| Yes | 0.81 (0.66-0.98) | 0.029 | 0.69 (0.52-0.92) | 0.012 |
| **AVT** | | | | |
| No | 1.00 (reference) | | 1.00 (reference) | |
| Yes | 1.33 (1.12-1.57) | 0.001 | 0.61 (0.49-0.75) | <0.001 |
| **Smoking history** | | | | |
| Non-smoker | 1.00 (reference) | | 1.00 (reference) | |
| Ex-smoker | 1.15 (0.97-1.37) | 0.11 | 1.03 (0.79-1.35) | 0.829 |
| Current-smoker | 1.15 (0.96-1.39) | 0.141 | 1.55 (1.18-2.03) | 0.002 |
| **Alcohol drink** | | | | |
| None | 1.00 (reference) | | 1.00 (reference) | |
| Moderate | 1.05 (0.90-1.24) | 0.527 | 0.92 (0.72-1.17) | 0.475 |
| Heavy | 1.01 (0.83-1.24) | 0.901 | 0.71 (0.52-0.98) | 0.035 |
| **Physical activity (METs-hour/week)** | | | | |
| 0 to < 3 | 1.00 (reference) | | 1.00 (reference) | |
| 3 to < 9 | 1.02 (0.86-1.22) | 0.812 | 0.74 (0.56-0.97) | 0.027 |
| 9 to < 18 | 0.99 (0.84-1.17) | 0.918 | 0.86 (0.68-1.10) | 0.231 |
| ≥18 | 1.13 (0.93-1.38) | 0.209 | 0.84 (0.63-1.13) | 0.247 |

Abbreviation: HR, hazard ratio; CI, confidence interval; HCC, hepatocellular carcinoma; MAFLD, metabolic dysfunction-associated fatty liver disease; HBV, hepatitis B virus

**Supplementary Table S4. Comparison of baseline characteristics according to MAFLD among patients with HBV-related HCC receiving surgical resection after PS-matching**

| Variables | PS-Matched Cohort (n=1440) | | |
|---|---|---|---|
| | non-MAFLD (n=720) | MAFLD (n=720) | p-value |
| **Age** | | | 1 |
| | 55.1 (8.7) | 54.8 (8.6) | |
| **Sex** | | | 0.754 |
| Male | 624 (86.7%) | 628 (87.2%) | |
| Female | 96 (13.3%) | 92 (12.8%) | |
| **Hypertension** | | | <0.001 |
| No | 474 (65.8%) | 372 (51.7%) | |
| Yes | 246 (34.2%) | 348 (48.3%) | |
| **Diabetes** | | | 0.019 |
| No | 538 (74.7%) | 498 (69.2%) | |
| Yes | 182 (25.3%) | 222 (30.8%) | |
| **Dyslipidemia** | | | 0.953 |
| No | 523 (72.6%) | 524 (72.8%) | |
| Yes | 197 (27.4%) | 196 (27.2%) | |
| **Liver cirrhosis** | | | 0.868 |
| No | 249 (34.6%) | 246 (34.2%) | |
| Yes | 471 (65.4%) | 474 (65.8%) | |
| **Metformin use** | | | 0.769 |
| No | 609 (84.6%) | 613 (85.1%) | |
| Yes | 111 (15.4%) | 107 (14.9%) | |
| **Statin use** | | | 1 |
| No | 606 (84.2%) | 606 (84.2%) | |
| Yes | 114 (15.8%) | 114 (15.8%) | |
| **Antiviral therapy** | | | 0.66 |
| No | 161 (22.4%) | 168 (23.3%) | |
| Yes | 559 (77.6%) | 552 (76.7%) | |
| **Smoking history** | | | 0.705 |
| Non-smoker | 276 (38.3%) | 261 (36.2%) | |
| Ex-smoker | 226 (31.4%) | 231 (32.1%) | |
| Current smoker | 218 (30.3%) | 228 (31.7%) | |
| **Alcohol drink** | | | 0.413 |
| None | 410 (56.9%) | 385 (53.5%) | |
| Moderate | 218 (30.3%) | 237 (32.9%) | |
| Heavy | 92 (12.8%) | 98 (13.6%) | |
| **Physical activity** | | | 0.263 |
| 0 to < 3 METs-hour/week | 239 (33.2%) | 236 (32.8%) | |
| 3 to < 9 METs-hour/week | 145 (20.2%) | 161 (22.4%) | |

| | | |
|---|---|---|
| 9 to < 18 METs-hour/week | 212 (29.4%) | 224 (31.1%) |
| ≥18METs-hour/week | 124 (17.2%) | 99 (13.7%) |

Abbreviation: MAFLD, metabolic dysfunction-associated fatty liver disease; HBV, hepatitis B virus; HCC, hepatocellular carcinoma; PS, propensity score

**Supplementary Table S5. Adjusted HRs and 95% CIs of HCC recurrence by FLI grade**

| | HCC recurrence | p-value | All-cause Mortality | p-value |
|---|---|---|---|---|
| **FLI grade** | | | | |
| Grade 0 (<30) | 1.00 (reference) | | 1.00 (reference) | |
| Grade 1 (30 to < 60) | 1.10 (0.94-1.28) | 0.243 | 1.27 (1.01-1.60) | 0.044 |
| Grade 2 (≥60) | 1.37 (1.13-1.65) | 0.001 | 1.82 (1.39-2.39) | <0.001 |

Abbreviation: HR, hazard ratio; CI, confidence interval; HCC, hepatocellular carcinoma; FLI, fatty liver index;

**Supplementary Table S6. The proportion of unhealthy lifestyle among individuals receiving health examination before or after the resection**

| | Men (n=1583) | | Women (n=449) | |
|---|---|---|---|---|
| | **before the resection (n=1240)** | **after the resection (n=343)** | **before the resection (n=336)** | **after the resection (n=113)** |
| **Smoking history** | | | | |
| Non-smoker | 334 (26.9%) | 113 (32.9%) | 306 (91.0%) | 107 (94.7%) |
| Ex-smoker | 375 (30.2%) | 185 (53.9%) | 12 (3.6%) | 5 (4.4%) |
| Current smoker | 531 (42.8%) | 45 (13.1%) | 18 (5.4%) | 1 (0.9%) |
| **Alcohol drink** | | | | |
| None | 529 (42.7%) | 302 (88.0%) | 269 (80.1%) | 110 (97.3%) |
| Moderate | 438 (35.3%) | 33 (9.6%) | 49 (14.6%) | 3 (2.7%) |
| Heavy | 273 (22.0%) | 8 (2.3%) | 18 (5.3%) | 0 (0.0%) |
| **Physical activity (METs-hour/week)** | | | | |
| 0 to < 3 | 415 (33.5%) | 76 (22.2%) | 115 (34.2%) | 35 (40.0%) |
| 3 to < 9 | 272 (21.9%) | 69 (20.1%) | 102 (30.4%) | 31 (27.4%) |
| 9 to < 18 | 386 (31.1%) | 109 (31.8%) | 75 (22.3%) | 33 (29.2%) |
| ≥18 | 167 (13.5%) | 89 (25.9%) | 44 (13.1%) | 14 (12.4%) |

Abbreviation: MET, metabolic equivalent

**Supplementary Table S7. The result of sensitivity analysis on the risk of HCC recurrence by MAFLD**

| Settings | adjusted HR (95% CI) | p-value |
|---|---|---|
| Individuals receiving health examination before surgical resection | 1.20 (1.02-1.40) | 0.026 |
| FLI cut-off ≥60 | 1.34 (1.13-1.60) | 0.001 |
| FLI cut-off ≥31 for men and ≥18 for women | 1.19 (1.05-1.36) | 0.009 |
| Without adjustment for metformin and statin | 1.18 (1.03-1.35) | 0.016 |
| Without patients receiving TACE within 3 months after surgical resection | 1.19 (1.04-1.37) | 0.013 |
| Without patients receiving additional RFA within 3 months after surgical resection | 1.20 (1.05-1.38) | 0.008 |

Abbreviation: MAFLD, metabolic dysfunction-associated fatty liver disease; HR, hazard ratio; CI, confidence interval; HCC, hepatocellular carcinoma

**Supplementary Figure Legends**

Figure S1. Detailed schematic flow of patient selection

Figure S2. Absolute standardized mean difference of the variables before and after matching
