# Supplementary figures and images for: Prognostic Impact of MAFLD Following Surgical Resection of Hepatitis B Virus-Related Hepatocellular Carcinoma: A Nationwide Cohort Study
Source: Cancers (Basel). 2022 Oct 13;14(20):5002. doi: 10.3390/cancers14205002 (PMC9599346; doi:10.3390/cancers14205002)

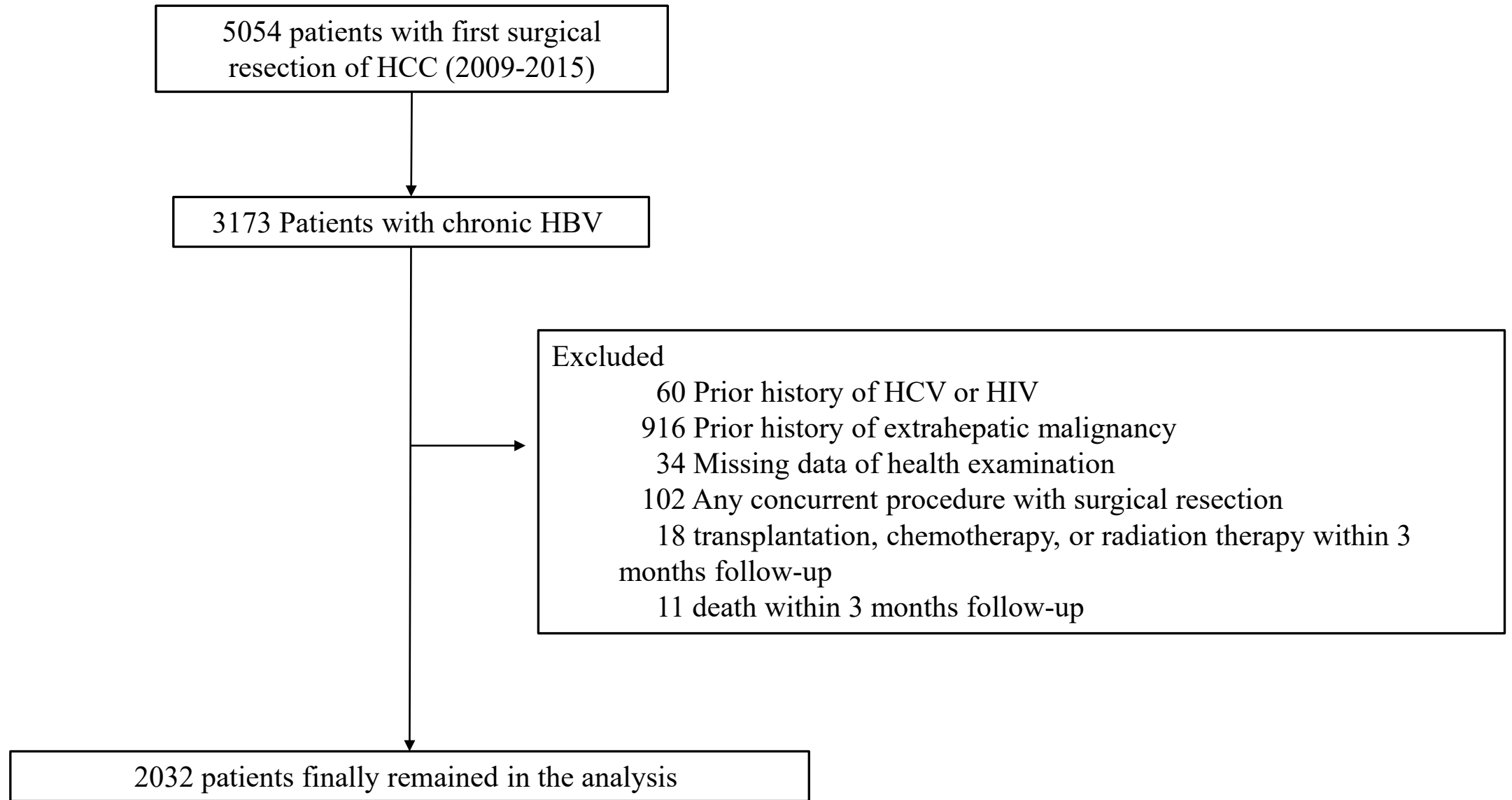

Supplement: Supplementary file 1 [file cancers-14-05002-s001.zip › figure_S1.pdf]

Absolute Mean Standard Deviation

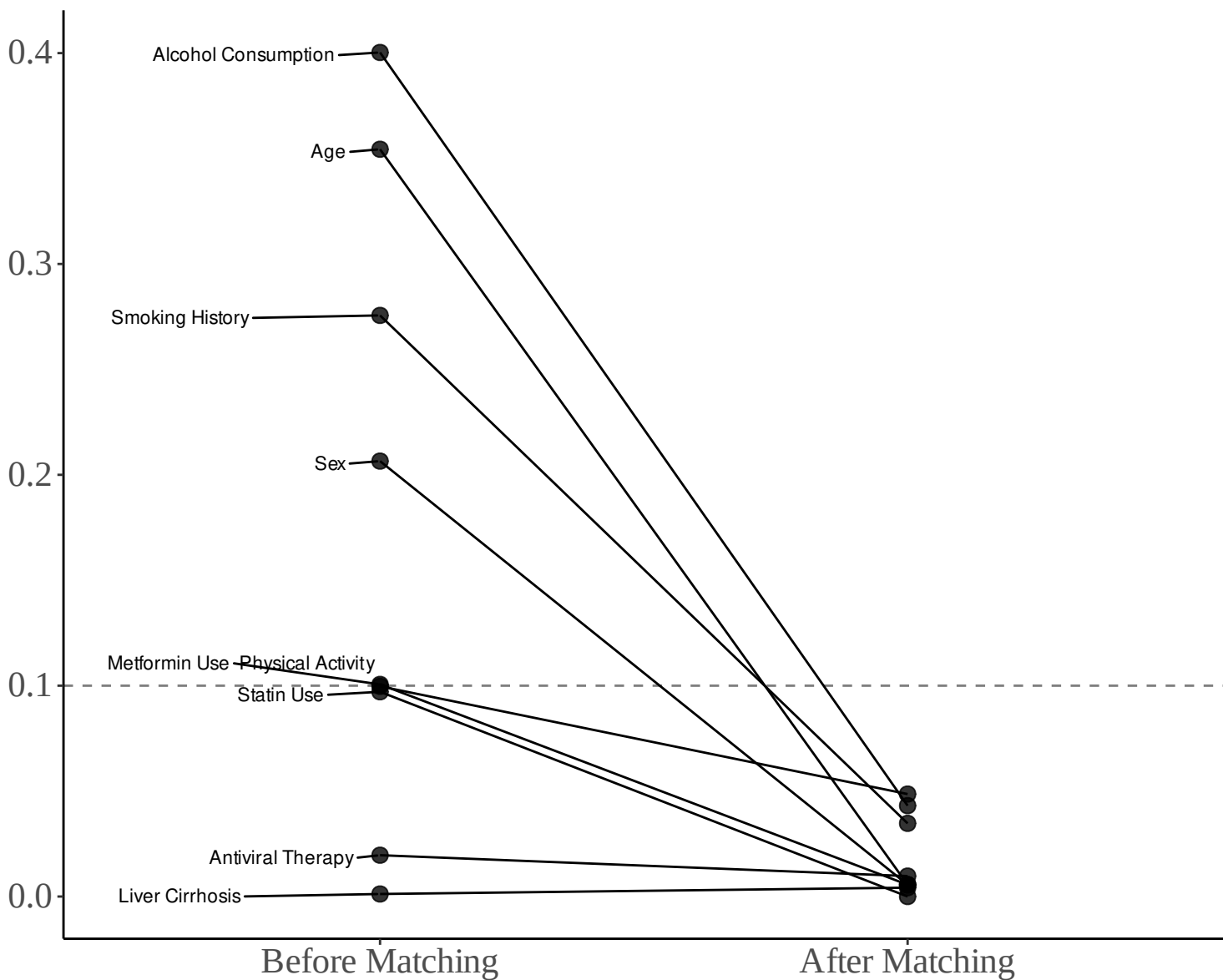

Supplement: Supplementary file 1 [file cancers-14-05002-s001.zip › figure_s2.pdf]
